# Changes in Lumbopelvic Movement and Muscle Recruitment Associated with Prolonged Deep Squatting: A Pilot Study

**DOI:** 10.3390/ijerph15051001

**Published:** 2018-05-16

**Authors:** Tim K. S. Lui, Sharon M. H. Tsang, Anthony W. L. Kwok

**Affiliations:** 1 Department of Physiotherapy, Tuen Mun Hospital, Hong Kong, China; lks27824819@hotmail.com; 2 Department of Rehabilitation Sciences, The Hong Kong Polytechnic University, Hong Kong, China; 3 School of Medical and Health Sciences, Tung Wah College, Hong Kong, China; anthonykwok@twc.edu.hk

**Keywords:** low back pain, prolonged squatting, lumbopelvic movement, kinematics, muscle recruitment, creep, workers

## Abstract

This study examined the changes in spinal kinematics and muscle recruitment of the lumbopelvic region associated with prolonged squatting. Eight subjects with chronic nonspecific low back pain (LBP) and eight asymptomatic subjects (AS) performed squat-to-stand and reverse movements, before and immediately after 15 min deep-squatting. Within-group and between-group differences in lumbopelvic kinematics and electromyographic activity acquired in lumbar erector spinae (ES), gluteus maximus (GM), and vastus lateralis (VL) were analyzed. During squat-to-stand after squatting, the LBP group showed slower then faster lumbar movement in the second and third quartiles, respectively. In the second quartile, the AS group moved with a significantly greater lumbar angle. However, significantly greater bilateral GM activity (+4–4.5%) was found in the LBP group only. A more profound decrease in bilateral ES activity (−10%) was also shown in the LBP group, yet this was nonsignificant compared to the AS group (−4%). In the third quartile, only the LBP group moved with a significantly greater lumbar angle, together with a significant increase in bilateral ES (+6–8%) and GM muscle (+2–3%) activity. The findings of the altered pattern of joint kinematics and recruitment of the key lumbopelvic muscles displayed in the LBP group inform on the possible mechanisms that may contribute to the increased risk of developing lumbar dysfunctions for people who work in prolonged squatting postures.

## 1. Introduction

It has been reported that approximately 42% of chronic low back pain (LBP) experienced by general workers were associated with various mechanical factors [1]. Working with the torso near the end of flexion range in a prolonged manner could be one of the most common working postures in various industrial workers, and it has been suggested to have contributions to the development of LBP [2]. Numerous working postures—for example, sitting, lifting objects from floor, and squatting—are associated with the end-range flexion of the trunk. Amongst these trunk flexion postures, deep squat, which involves flexion of both the lumbar spine and hip joints to their end of ranges, contributes to a substantially greater degree of total body flexion compared to sitting and forward bending in standing. Previous studies have indicated that prolonged squatting was attributed as one of the significant risk factors of development of LBP in various occupations [3,4,5]. It has been shown that kneeling and squatting were associated with LBP in industrial workers with an alarming odds ratio of 4.62. As high as 92% of regular farm workers and 89% of wind farm workers experienced LBP associated with their high-demand job from working in the deep-squatting posture for a prolonged period of time. A study revealed as high as 60% of a cohort of individuals who engaged in regular gardening work with the adoption of deep squatting reported to have experienced back pain during and/after their gardening work [6].

There is substantial evidence reported in various mechanistic studies to partly explain the potential risks associated with adoption of end-range flexion postures [7,8]. Spinal stability is regarded as relying on the integrity and coordination among the (a) active subsystem (e.g., muscles and tendons); (b) passive subsystem (e.g., vertebrae, discs, and ligaments); and (c) neural subsystem (nerves and central nervous system) [9,10]. Therefore, presence of deficiency in any of these subsystems may trigger the compensation of complementary subsystem(s) within the spinal stability model. Flexion–relaxation phenomenon—a clinical presentation which is characterized by the cessation of the muscle activity at the lumbar erector spinae when an end-range lumbar flexion posture is adopted—may predispose the posterior spinal tissues, such as thoracolumbar fascia and spinal ligaments, to a higher level of stretch effect [11]. It is interesting to note that this myoelectric silence was found to be either less prevalent or even absent in individuals with low back pain [11,12]. The decrease or cessation of back extensors’ activity at end-range flexion may ultimately lead to increase in creep of the viscoelastic tissues and decrease the reflexive muscular activation of the back extensor muscles [13]. A previous study has also found that fatiguing of the low back extensor muscles occurred during and after prolonged deep trunk flexion in association with the reduction in motor unit activation, average conduction velocity, and force generating capacity due to prolonged passive stretch effect [14]. These changes may have an impact on the movement trajectory of the lumbar spine in response to the adoption of prolonged flexion posture. Creep of the spinal tissues has been reported to have occurred after 30–60 min deep trunk flexion in healthy subjects [15,16]. This creep effect of the posterior spinal soft tissues could be more profound and long lasting in workers who have much greater exposure to frequent and/or prolonged squatting due to their job nature. In order to reduce this occupational risk, comprehensive understanding of the possible changes in the lumbopelvic movements and muscle recruitment pattern associated with prolonged flexion or squatting position would be of great importance to help unravel the link between the associated impact on the lumbar spine and development of LBP.

There is only one study that has examined the angular displacement of lumbar spine and hip joint, where subjects executed the stand-to-squat activities consecutively for five repetitions [17]. Their findings showed a significantly smaller lumbar flexion angle and greater hip flexion angle in the LBP group during squatting compared to those who were asymptomatic. However, in-depth understanding of the potential risks associated with prolonged squatting still remains limited because only the immediate kinematics of hips and lumbar spine in the static squatting position were studied, without examining the impacts on the dynamic movement of lumbar spine and hip joints during the standing-up movement after prolonged squatting.

To fill this identified knowledge gap, this study aimed to examine the immediate effects of prolonged deep squatting on the dynamic movement pattern and muscle recruitment of the lumbar spine and hip joints. In addition, these effects were different between individuals with and without LBP. It was hypothesized that there were significant between-group differences in the lumbopelvic movement and muscle recruitment pattern immediately after adoption of the prolonged deep squatting posture. Findings of the present study would help promote the understanding of the risks associated with this specific working posture, which workers from different industries have to adopt frequently in their workplaces.

## 2. Materials and Methods

### 2.1. Study Design

This was a comparative cross-sectional study. Two groups of subjects, those presented with chronic nonspecific LBP (LBP group) and those who were asymptomatic (AS group), were included in this study. Assessments of their lumbopelvic kinematics (angular displacement of the lumbar spine and hip joints) and muscle recruitment pattern were conducted before and immediately after an experimental static postural adoption protocol, namely, the prolonged deep squatting position. This study complied with Declaration of Helsinki and Good Clinical Practice guidelines by International Conference on Harmonization of Technical Requirements for Registration of Pharmaceuticals for Human Use, and ethic approvals have been attained from The Chinese University of Hong Kong (CREC 2016.703) and The Hong Kong Polytechnic University (HSEARS2016004002).

### 2.2. Subjects

A total of 16 adults (all males) were recruited from the local community and local university, with 8 subjects each in both groups: the LBP group (*n* = 8) and the AS group (*n* = 8). Group demographics are presented in Table 1. Inclusion criteria for subject recruitment of the LBP group included: individuals presented with recurrent LBP (without referred symptoms beyond their gluteal folds) which lasted for ≥3 months; back pain without history of trauma and specific diagnosis; and with no or minimal intensity of LBP, rating ≤2 out of 10 on the visual analogue scale from 0–10 (VAS 0–10). For recruitment of the AS group, age-matched individuals without history of LBP over the previous 12 months were recruited. Subjects in both groups were excluded if they have any history of surgery or structural deformity of their spine or lower limbs, injury or pain in their lower limbs which affected their capacity to perform/tolerate deep squatting, any known neurological or metabolic disease, history of dizziness, or postural hypotension or its associated symptoms.

Detailed explanation of the study was given to each subject, and written informed consent was obtained prior to the study. Subjects were requested not to engage in any heavy physical activities and training 24 h before the assessment [18]. All assessments were carried out between 2 p.m. and 8 p.m. consistently by one assessor at the university laboratory, and standardized instructions were used for all assessment and measurement procedures.

### 2.3. Measurements

**Figure 1:**
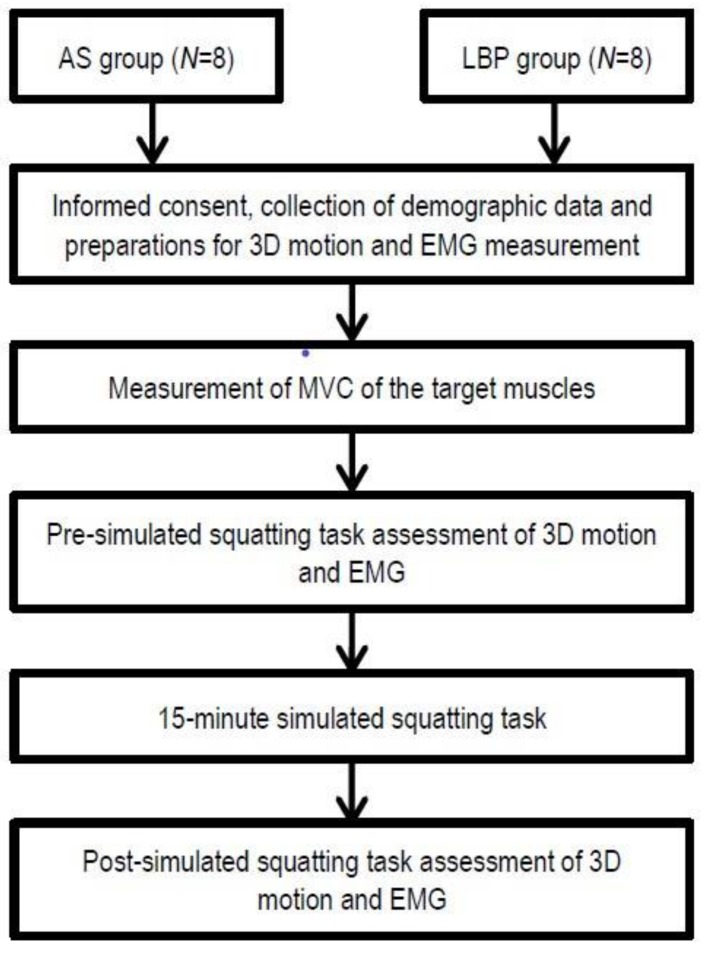
Experimental procedures. Notes: LBP: low back pain. AS: asymptomatic; MVC: maximal voluntary contraction; EMG: Electromyography.

Lumbopelvic movement and muscle recruitment pattern when performing the squat-to-stand and its reverse action were measured before and immediately after a 15 min deep-squatting position (Figure 1). The detailed procedures and the instrumentations are described in the following Section 2.3.1, Section 2.3.2, Section 2.3.3 and Section 2.3.4. These biomechanical data were compared within groups and between two groups for analysis.

#### 2.3.1. Measurement of Joint Kinematics

**Figure 2:**
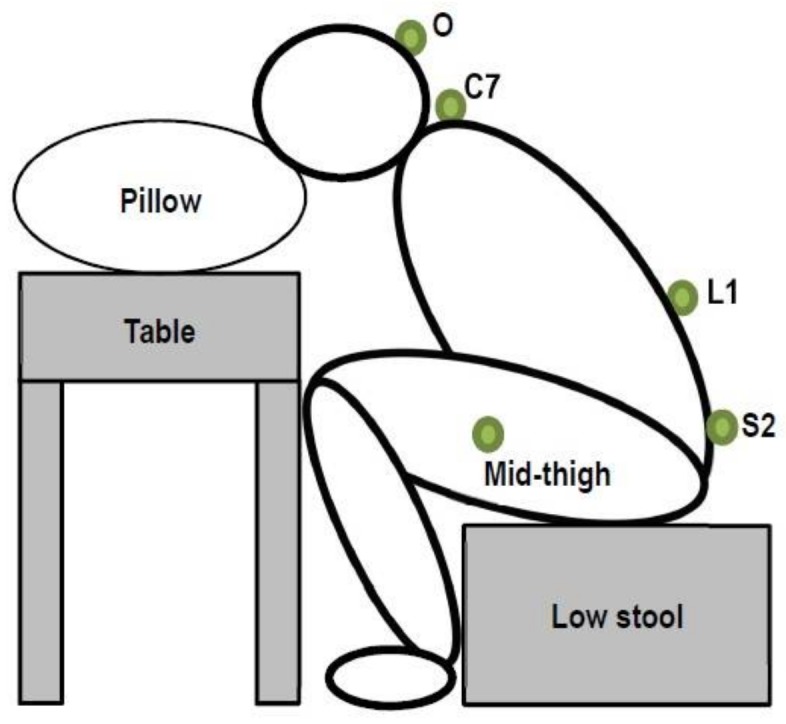
Simulated deep squatting position. Notes: O = occiput, C7 = spinous process of C7 vertebra, L1 = spinous process of L1 vertebra, S2 = spinous process of S2 vertebra.

Three-dimensional kinematics of the spine and lower limbs was recorded by the compact wireless inertial measurement units (IMU) (MyoMotion, Noraxon USA, Inc., Scottsdale, AZ, USA) at a sampling frequency of 100 Hz. The joint range of motion (ROM) was determined by positioning individual IMUs on two contiguous body segments. In this study, joint ROM at the sagittal plane would be the main focus for the movement to be examined. To measure the lumbar spine and hip joint angles, four IMU sensors were attached onto the spinous processes of L1 and S2 vertebrae and lateral side of both thighs, midway of the line connecting the lateral epicondyle and greater trochanter of the femur, respectively (Figure 2). All sensors were secured by double adhesive tapes and further stabilized using sports tape to minimize relative movements between the sensors and the underlying skin [19,20]. Two additional sensors were placed over the external occipital protuberance and the spinous process of the C7 vertebral level in order to enable the real-time monitoring of the head and thoracic spine range during the deep squatting position adoption. The joint angles of both hips were computed based on the differential signals acquired from the sensors placed over the thigh of the respective side and spinous process of the S2 vertebral level, while lumbar angle was the differential signal acquired from the sensors at the spinous processes of L1 and S2 vertebral level [19]. The IMU system has been found to have <10% of coefficient of variation, which indicates stable and reliable measurements of motion data. Its concurrent validity has been established with the optoelectronic system and the correlation coefficients *(r*^2^) related to the measurement of trunk movement ranged from 0.94 to 0.99, as reported in previous study [21].

#### 2.3.2. Measurement of Muscle Recruitment

Muscle recruitment pattern was examined using the electromyography (EMG) activity measured from three pairs of muscles over the lumbopelvic region using the MyoMuscle 1400A 16-channel Fixed-Cable surface EMG system (Noraxon USA Inc., Scottsdale, AZ, USA). The three pairs of muscles included the lumbar erector spinae (ES), vastus lateralis (VL), and gluteus maximus (GM). The EMG signals were recorded at a sampling frequency of 1000 Hz and the data acquisitions of the kinematics and EMG were synchronized. Standardized skin preparations for surface EMG measurement, which included removal of hairs and light abrasion of skin by sandpaper followed by cleaning with alcohol swabs, were applied and the skin impedance was monitored by the impedance meter at level ≤5 kΩ. Disposable bipolar EMG Ag/AgCl electrodes were positioned with the interelectrode distance of 2 cm over three pairs of spinal and lower limb muscles by following the standardized surface EMG electrode placement recommended by Surface Electromyography for the Non-Invasive Assessment of Muscles (SENIAM) projects [22]. Specifically, EMG electrodes were placed over: (a) bilateral lumbar ES as two fingers width lateral from the spinous process of L1 vertebral level; (b) midpoints of the line connecting the sacral vertebrae and greater trochanter on bilateral GM; (c) two-third of the line connecting anterior superior iliac spine and tip of lateral border of patella on bilateral VL [23]. Adhesive tapes were used for fixation of electrodes and cables to the skin, and the ground reference electrode was placed on the spinous process of the L4 vertebral level of subjects.

#### 2.3.3. Measurement of Maximal Voluntary Contraction (MVC)

Before the baseline measurement of the movement and muscle recruitment pattern, the MVC of the three muscle pairs was measured. Three trials of MVC testing for each muscle were carried out, with 2 min rest interval between trials. The largest amplitude of EMG across each trial was identified for a normalization procedure of muscle activity in the respective muscles. Subjects were asked to contract the targeted muscle group against the manual resistance applied by the assessor, as hard as they could for 5 s consecutively [24]. For the MVC of ES, resisted trunk extension was performed with the subject in prone-lying position with their hands placed on their forehead and their legs stabilized by the strap at mid-calf level [25]. For the MVC of GM, resisted unilateral hip extension was performed with the subject in prone-lying position with the contralateral leg stabilized [25]. For the MVC of VL, resisted unilateral knee extension was performed by the subject in sitting position with their hips positioned at 90° flexion and the trunk stabilized firmly to minimize contributions of effort from muscles other than the targeted ones [26].

#### 2.3.4. Simulated Deep Squatting Position and Assessments of Squat-to-Stand and Stand-to-Squat Tasks

**Figure 3:**
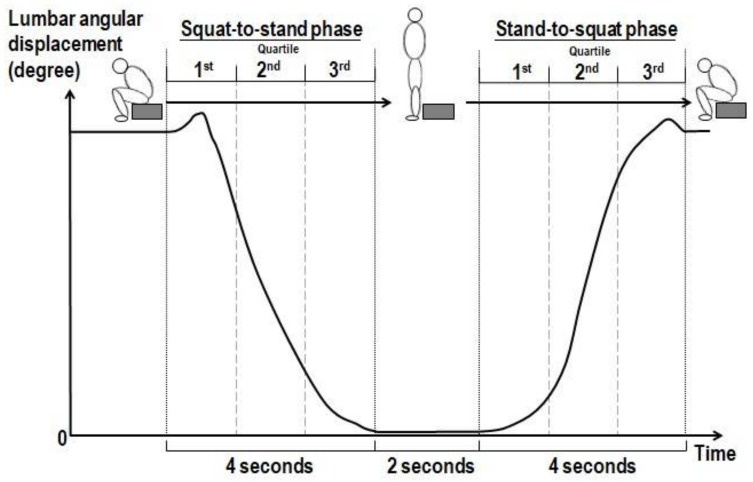
Procedures of execution of squat-to-stand and stand-to-squat tasks (performed before and immediately after deep squatting).

After the MVC procedure, a baseline assessment of the lumbopelvic movement and muscle recruitment during the squat-to-stand and stand-to-squat tasks was carried out. Subjects were asked to adopt the experimental position that simulated the deep squatting posture (Figure 2 and Figure 3).

They were guided to adopt a deep sitting position with the stool height adjusted to 50% of their tibial length (distance measured between lateral knee joint and medial malleolus of tibia). To standardize the deep squatting position, subjects were required to sit on the stool padded with a cushion of 2 inches thick, with their knees at 130° flexion. Their feet were positioned at 20° external rotation from midline with their Achilles tendons in contact with the front edge of the stool. This specific foot position was marked with footprints placed on the floor for standardization in the repeated assessments of the squat-to-stand and reverse action after the simulated deep squatting position. To ensure the achievement of the end-range positioning of the lumbar flexion and hip joints, subjects were instructed to have their trunk slouched as much as possible until their abdomen was in contact with their thighs. With the deep squatting position properly adopted, subjects were first asked to stand up without using their upper limbs for support from the deep squatting position (defined as squat-to-stand phase) in 4 s without hand support, then to remain in their upright standing position for 2 s before finally sitting down onto the low stool (defined as stand-to-squat phase) in 4 s (Figure 3). They were allowed to practice the tasks a few times before the actual data collection in order to get themselves familiar with the tasks they were asked to perform. The time of each phase was standardized to minimize the potential confounding effect related to the differences in the execution time between subjects and between trials. Three trials were repeated for each subject as the baseline assessment.

After the baseline assessment, the subjects were required to adopt the standardized deep squatting position for 15 min continuously to simulate the prolonged deep squatting at the workplace. In addition to the standardization of the lumbar spine and hip joint angle specified above, confirmation of subjective feeling of tension or stretching feeling experienced by each subject was also obtained, together with the continuous monitoring of the EMG activity level of the bilateral ES during the static posture period. To minimize stress to the neck region during the 15 min of deep squatting, subjects were instructed to have their heads and forearms rested on a pillow placed over the supported surface located in front of them. In regard to the main objective of this study, the simulated squatting position was thoroughly planned to balance the achievement of possible creep effect at the tissues over the lumbar spine and to minimize any unnecessary discomfort or stress to their lower limbs and neck region. Blood pressure was measured continuously throughout the 15 min of static postural adoption in order to monitor development of potential dizziness associated with postural hypotension. The degree of discomfort and/or pain experienced over the lumbar region of the subjects was monitored throughout the whole 15 min period. The experiment would have been terminated if subjects reported experiencing any pain or discomfort in any body parts other than over their lower back region, or if the intensity of their back pain increased more than 2/10 points, on the 10-point VAS, from their baseline level.

Upon the completion of the 15 min of deep squatting, the kinematics and muscle recruitment of the lumbopelvic region were reassessed during the squat-to-stand and stand-to-squat tasks using the same procedures described. To ensure the safety and comfort of all subjects, subjects were required to take a rest for a minimum of 10 min at the laboratory after the completion of all assessment procedures before they left.

#### 2.3.5. Data Processing and Statistical Analysis

The kinematic data and EMG data were standardized to 10,000 data points, and data points in each phase were then equally divided into three quartiles (the first, second, and third quartiles) (Figure 3). The reliability of the data measured in the three trials of task performance was assessed using the intraclass correlation coefficient analysis (ICC). The angles of the lumbar spine and bilateral hip joints during the deep squatting position were compared between the two groups using the independent *t*-test. EMG signals were full wave rectified, bandpass-filtered at 20–300 Hz, and smoothed at 50 ms moving windows to produce the linear envelope of the root-mean-square (RMS). The EMG amplitudes were then quantified to percentage of EMG activity obtained during the MVC procedure of the respective muscles, expressed in percentage of MVC (%MVC) for comparisons. Mean value of the change in EMG activity level of the six muscles and hip and lumbar flexion angles (with the post-data minus the pre-data) in each of the three quartiles for each task phase was computed for statistical analysis. Positive values of the changes indicate an increase in the regional angle (expressed in degree) or increase in muscle activity (expressed in %MVC) at post-assessment, respectively.

The SPSS software 23.0 (IBM SPSS, Inc., Armonk, NY, USA) was used to analyze the differences of change in angles and EMG activity before and after 15 min squatting within-group by paired *t*-test, or Wilcoxon signed-rank test if the criterion of normality was not fulfilled. Data were further analyzed to determine the interaction between time-and-group using two-way repeated measures ANOVA. The level of significance was set at 0.05.

## 3. Results

Initially, only 1 subject in the LBP group had LBP rated as 0.5 out of the 10-point VAS before the baseline assessment, and 4 of the 8 subjects (i.e., 50%) in the LBP group developed the usual pain over their lower back (at most 2 out of 10 on VAS) during 15 min squatting. All subjects reported to have their back pain completely subsided after completing the final set of squat-to-stand and stand-to-squat tasks. No report of discomfort over other body parts from subjects occurred throughout the study. No latent pain was reported from subjects after a 10 min rest period upon completion of all assessments.

### 3.1. Standardization and Verification of Simulated Deep Squatting Position

There were no significant differences in lumbar, hips, and total flexion angle in simulated squatting position between groups (Table 2). Table 3 shows the EMG activity in terms of the percentage of MVC of ES muscles of all 16 subjects during the simulated squatting task. There were 25 out of 32 ES muscles having less than 5% MVC of the ES during the task, suggesting validity of creep model at the simulated squatting position [27].

### 3.2. Trajectory of Regional Joint Angles of the Lumbopelvic Region during Squat-to-Stand and Stand-to-Squat Tasks

#### 3.2.1. During Squat-to-Stand Phase (Before and Immediately after Deep Squatting)

**Figure 4:**
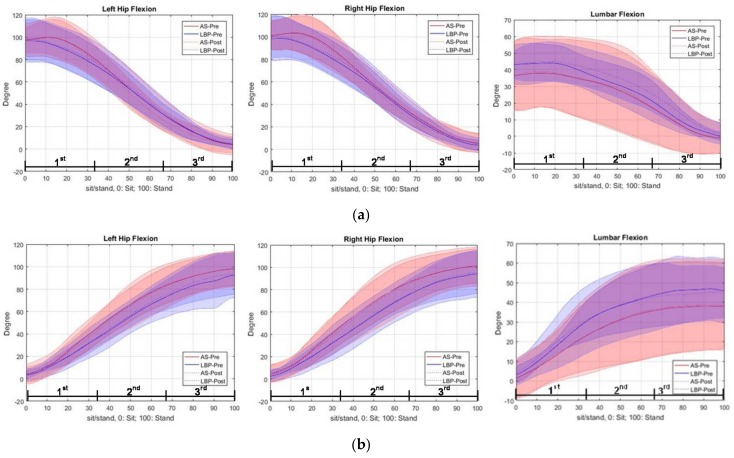
Trajectories of lumbar spine and hip joint angles (mean ± 1SD) during (**a**) squat-to-stand phase and (**b**) stand-to-squat phase, before and immediately after squatting. The mean values of the pre-squatting performance (solid lines) and post-squatting performance (dotted lines) are presented with the red lines for the AS group and the blue lines for the LBP group.

Both hips in the AS group had a greater gradient of trajectories in the second and third quartile of movement in the post-task assessment than that in the pre-task, while the gradients were similar, in general, before and after the task in the LBP group (Figure 4a). For lumbar spine angle, the LBP group had a smaller gradient of trajectory in the second quartile and a greater gradient in the third quartile of movement of the post-task assessment than that of pre-task. For the AS group, the gradient of lumbar spine angles was similar in the movement before and after the task.

#### 3.2.2. During Stand-to-Squat Phase (Before and Immediately after Deep Squatting)

There was no pre–post difference found between groups, in general, for the trajectory of hip joint angles bilaterally (Figure 4b). For lumbar spine angle, the gradient of trajectory was generally less steep in the LBP group in the movement after the task, but it was similar in the movement before and after the task in the AS group.

#### 3.2.3. Changes in Lumbar Spine and Hip Joint Flexion Angles

**Figure 5:**
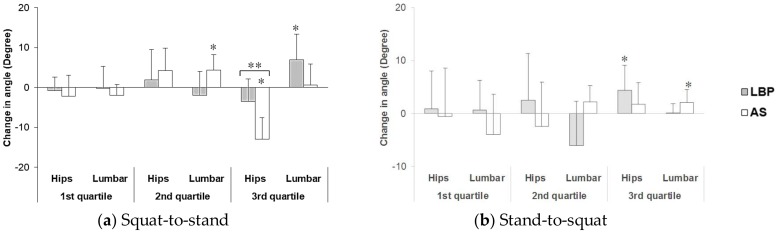
Changes in lumbar spine and hip joint angles (mean ± 1SD) before and immediately after 15 min squatting during (**a**) squat-to-stand and (**b**) stand-to-squat phase. Positive values of the changes indicate an increase in the regional angle (expressed in degree) at post-assessment compared to pre-assessment. * Indicates significant within-group differences and ** indicates significant time × group interaction.

Figure 5 shows the change in lumbar and mean change in hip angles in the squat-to-stand and stand-to-squat tasks before and after the adoption of 15 min deep squatting, with all data having interclass correlation (ICC) above 0.8 among three trials.

- During Squat-to-Stand Phase

In the first quartile, there was no significant difference in the change in lumbar angle and mean change in hip angles between the pre- and post-task assessments. In the second quartile, the AS group showed a significant increase in change in lumbar angle (*p* = 0.017) at the post-task assessment. In the third quartile, the AS group showed a significant change (decrease) in mean hip angle (*p* < 0.001) at post-task assessment. There was a significant time × group interaction effect (*p* = 0.004), revealing a significant between-group difference in the changes of hip angle in the third quartile, in response to prolonged deep squatting. In addition, the LBP group showed a significant change in lumbar angle (increase) (*p* = 0.019) in the third quartile, at the post-task assessment.

- During Stand-to-Squat Phase

In both the first and second quartiles, changes in lumbar angle and mean change in hip angles showed no significant pre–post difference in both groups. In the third quartile, the LBP group showed a significantly increased mean hip angle (increase) (*p* = 0.035), while the AS group showed a significant change in lumbar angle (increase) (*p* = 0.039) at the post-assessment.

### 3.3. Muscle Recruitment Pattern (Expressed in %MVC of EMG Activity)

**Figure 6:**
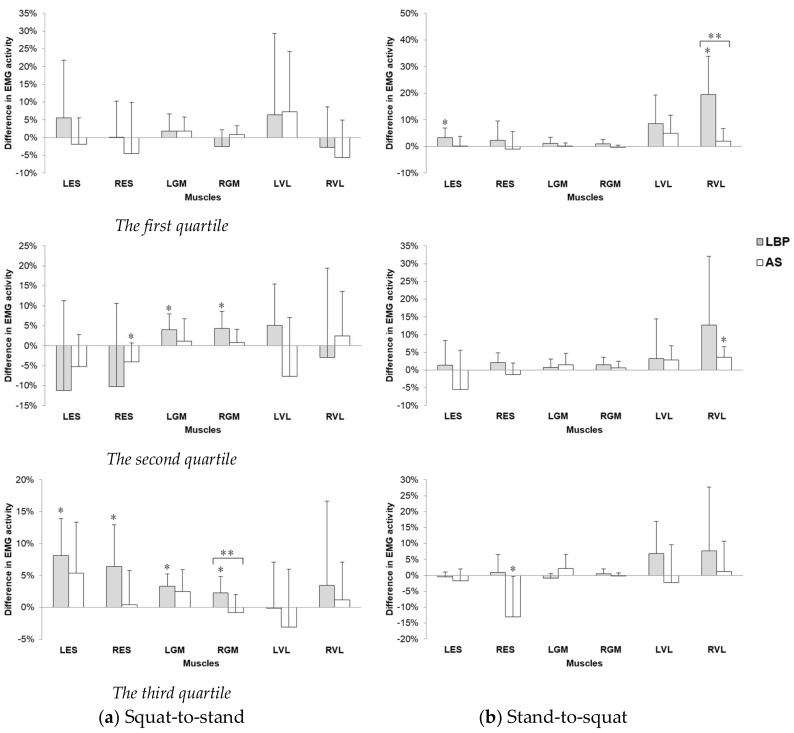
Changes in EMG activity of muscles (mean ± 1SD) before and immediately after 15 min deep squatting at the three quartiles during (**a**) squat-to-stand phase and (**b**) stand-to-squat phase. L = left, R = right, ES = erector spinae muscle, GM = gluteus maximus muscle, VL = vastus lateralis muscle. Positive values of the changes indicate an increase in muscle activity (expressed in %MVC) at post-assessment compared to pre-assessment. * Indicates significant within-group differences and ** indicates significant time × group interaction.

Figure 6 shows the EMG activity of related muscles in squat-to-stand and stand-to-squat phases before and after prolonged deep squatting, with all data having ICC values above 0.8 among three trials.

#### 3.3.1. During Squat-to-Stand Phase

In the first quartile, there was no significant difference found in the change in muscle activity of all the six muscles between the pre- and post-task assessment. In the second quartile, there was a decrease in bilateral ES muscle activity in the post-task assessments by about 10% and 4% in the LBP and AS group, respectively. However, only the decrease in muscle activity of right ES muscle after 15 min squatting in the AS group showed statistical significance (*p* = 0.048) in post-task assessment compared to that in pre-task. For GM muscles on both sides, there was significant increase in GM muscle activity by 4–4.5% in the movement after squatting in the LBP group (left side: *p* = 0.025, right side: *p* = 0.022) but not in the AS group, and there was no significant pre-post difference in bilateral VL muscle activity after the task. In the third quartile, only the LBP group had a significantly higher bilateral ES muscle activity in the movement by 6–8% after prolonged squatting (left side: *p* = 0.005, right: *p* = 0.028) than that before. With no significant difference in muscle activity in the pre-task assessment, there was nearly significant time × group interaction in right ES (*p* = 0.066) only. In bilateral GM muscles, the LBP group had significantly higher muscle activity by 2–3% in post-task assessment than that in the pre-task assessment (left side: *p* = 0.002, right: *p* = 0.039), but not in the AS group, with only significant time × group interaction (*p* = 0.038) in right GM muscles. In bilateral VL muscles, there was no significant difference in muscle activity before and after 15 min squatting.

#### 3.3.2. During Stand-to-Squat Phase

In the first quartile, there was significant increase in muscle activity of left ES muscle (*p* = 0.043) and right VL muscles (*p* = 0.007), and nearly significant increase in muscle activity of left VL muscle activity (*p* = 0.058) in the movements after 15 min squatting in the LBP group than that before the task, but not in the AS group, and only that in right VL muscle showed significant time × group interaction (*p* = 0.006). In the second quartile, only the AS group showed a significant increase in muscle activity (*p =* 0.013) over right VL muscles, but there was a nearly significant difference in muscle activity (*p* = 0.079) over left VL muscles in the movements after 15 min squatting than that before, but not in the LBP group, with no significant time × group interaction. In the third quartile, the AS group had a significant decrease in muscle activity (*p* = 0.023) over right ES muscle in post-task assessment than that of pre-task, but not in the LBP group.

## 4. Discussion

### 4.1. Verification of Deep Squatting Position

This study examined the immediate effect of prolonged deep squatting on the lumbopelvic kinematics and muscle recruitment using the simulated deep squatting position, in which stress to lower limbs was reduced by having to support their body weight over their buttocks. A previous study suggested that curvature of the spine would alter both the location and magnitude of load on the spinal tissues [28]. Therefore, variation of the spinal curvature during deep squatting position in this study, if present, would have influenced the load sharing within the spinal tissues. However, with the comparable amount of regional and total flexion angles verified during the 15 min deep squatting position between two groups, these possible confounding effects have been minimized. Furthermore, the activity level of the bilateral back extensors (ES) in most of subjects (25 out of 32 ES muscles) have achieved the flexion–relaxation status during simulated squatting position, in which the ES activity level was <5% of MVC of ES muscles in lumbar flexion position. This specific level of ES activity was set as an integral criterion to indicate the presence of flexion–relaxation response in a previous study [29]. In addition, all subjects affirmed the presence of tissue stretch feeling over their lower back developed in the beginning of simulated squatting position, further reinforcing the validity of attaining the end-range body flexion position and possible muscle creep in the ES muscles during the simulated deep squatting position. In contrast to the previous report of absence of flexion–relaxation phenomenon at the lumbar extensors in people with chronic low back pain [12], the presence of the substantial reduction of the activity levels found in this present study could be explained by the differences in the time duration for which the lumbar spine was positioned at its end of range between studies.

In contrast, restriction of lumbar flexion and compensation of having greater flexion at the hip joints during squatting in individuals with LBP compared to able-bodies, reported by Sung et al. [17], were not observed in the present study. Significant changes in the lumbopelvic movement kinematics and muscle recruitment were present in both symptomatic and healthy control groups, immediately after the simulated deep squatting position. The differences in the manifestations of those changes revealed between two groups are further discussed below.

### 4.2. Changes in Movement Kinematics and Muscle Recruitment after Adoption of Prolonged Deep Squatting Posture

The deep squatting position caused a delayed lumbar extension from the second quartile to the third quartile of the squat-to-stand phase in subjects with LBP (as shown in Figure 4a). After the prolonged squatting, the lumbar extension in the AS group mainly took place during the second quartile. Meanwhile, an associated reduced hip movement was observed in the healthy group, but not in the LBP group. During the squat-to-stand phase, both the lumbar spine and hip joints are considered to be moving from a fully flexed posture to a neutral degree of extension (when the subject reaching the upright standing posture at the end of this phase). Tissues located at the dorsal aspect of the body would move from their lengthened position to shortened position. The presence of the flexion–relaxation phenomenon associated with the deep squatting position appears to have a substantial negative impact on the activation of the lumbar extensors. This hypothesis was supported by the decrease in EMG amplitude of the bilateral ES in the second quartile but significant increase of their activity level in the third quartile, found at the deep squatting position. In addition, a modified recruitment pattern was also observed over the primary hip extensors during the second and third quartiles of the squat-to-stand movement at post-squatting assessment. The delay of the lumbar spine in restoring its neutral position from full flexion displayed in the LBP group could be explained by the plausible deformation of the viscoelastic tissues at the posterior back region and decrease in reflexive muscular activation of the erector spinae muscles [2,30]. Such relaxation or even cessation of erector spinae muscles related to sustained end-range stretching of the tissues has been identified as a key predisposing factor for lower back injury [11,31,32]. It is important to highlight that despite the contrasting response of lumbar kinematics observed in healthy controls, a similar negative effect of ES recruitment was also found in their right back extensor muscles. A few factors might have contributed to this particular finding. First, the 15 min deep squatting task might not cause the same level of tissue creep in both groups. As reported by Abboud et al. [15], longer duration (e.g., 30–60 min) may be required for the tissues to achieve the creep effects in those healthy individuals. Despite the less profound changes observed here, the findings still indicate potential risk of back injury related to prolonged adoption of body flexion posture. Besides the deleterious impact on the back extensors attributed to the inhibited reflexive muscular activation, the development of creep effect of the posterior spinal tissues (e.g., thoracolumbar fascia and spinal ligaments) would ultimately lead to fatigue of these muscles [14]. These combined physiological effects have consistently been found to be contributing factors to development and/or aggravation of LBP [33,34].

Overall, changes in both the regional kinematics and EMG amplitudes were less apparent during the stand-to-squat phase compared to the squat-to-stand phase at the post-assessment. A different pattern was found in which the LBP group had greater increase in hip angle, while the AS group had greater lumbar angle, when they were in the late stage of squatting. However, no between-group differences were found in the movement pattern. Changes in response to the deep squatting were mainly found over the lumbar and knee extensor muscles in the LBP group. Significant increases in the left ES and right VL (in the first quartile) and left VL (in the second quartile) were shown when the symptomatic subjects moved from upright to squatting position, after the deep squatting position adoption. A significant between-group difference was found in right VL in the first quartile, which suggests that individuals with LBP engaged greater eccentric knee extensor work when they lowered themselves from upright standing. Different to the previous report of quadriceps inhibition after repeated lumbar extension task in both the healthy and LBP groups of adults [35], enhanced recruitment of the quadriceps (i.e., VL activity in this study) was displayed in the LBP group throughout the initial and middle ranges of eccentric quadriceps contraction. It is possible that greater muscle efforts from large muscle groups (e.g., quadriceps) may be one of the compensatory strategies adopted in some individuals with LBP. However, more in-depth investigations may be necessary to confirm this postulation. The mechanisms underlying such variations of muscle recruitment pattern require further work that includes studying all the other muscles that are relevant to the muscle synergy system to better unravel the complexity of the neuromuscular adaptations.

Regarding the reciprocal GM and ES muscle recruitment shown in the LBP group during the squat-to-stand phase of movement in response to prolonged deep squatting, greater contribution to effort from the bilateral GM could be required to support the execution of the stand-up task for the negative effects on the back extensors associated with creep of the soft tissues and decrease in the muscle activity. A systematic review published by Wilson and her colleagues highlighted the important role of the gluteus maximus in rehabilitation of lumbar spine and lower limb injury based on the alterations to the function of the kinetic chain [36]. Changes in the GM recruitment pattern and impaired lumbopelvic movement rhythm has been associated with chronic LBP [37,38]. Incorporating GM strengthening and endurance exercise as the rehabilitation strategy for LBP has been proposed to maximize the clinical recovery [36].

### 4.3. Limitations and Recommendations

Several limitations have been identified in this study. There were only 16 young adult males recruited in this study, and subjects in the LBP group belonged to the subclinical back pain category. The present findings would therefore be confined to the age and LBP subgroup specified here. To improve the power and generalizability of this study, further work with the same experimental procedures and recruitment of a large cohort of subjects with corresponding age ranges is recommended. This critical comment is made with reference to the effects of age and gender differences on the flexion angle and lumbar curvature on the lumbopelvic rhythm during lumbar forward flexion reported in previous study [39]. In addition, it was only the primary extensor group of muscles over the lumbar spine and hip and knee joints being examined in this study. Other major muscles of the deep abdominal core muscle group (e.g., transversus abdominus and lumbar multifidus) should be included by considering the application of intramuscular EMG (iEMG). However, the practical limitations in accessing these deep layers of muscles using the iEMG method during dynamic movement of the trunk need to be addressed for the risk of needle dislodgement when performing isotonic muscle contraction and/or movements [40].

This study discussed the potential risks of prolonged deep squatting for symptom aggravation in individuals with chronic LBP, as well as for development of LBP in healthy individuals. Workers who are required to adopt deep squatting in a frequent and prolonged manner (e.g., repair workers, dishwashers, construction site workers) may become more susceptible to those risks, and optimal safety measures should be implemented. Previous studies have suggested that pelvic forward tilt during squat-to-stand movement might be one of the effective preventive methods for LBP in the workplace. It could lead to greater ES activities for adequate muscular support for the spine [41] and decrease compression and shearing force acting on the lumbar spine during squat lifting [42]. These were particularly important after prolonged squatting for the impact of the flexion–relaxation response of ES muscles and posterior spinal tissue creep previously discussed.

## 5. Conclusions

Individuals with chronic nonspecific LBP of mild degree showed significant differences in neuromuscular adaptations at the lumbopelvic region in response to prolonged squatting posture. Compared to those who were asymptomatic, individuals with LBP displayed modifications of their lumbopelvic movement pattern, with a significant increase in their lumbar angle and greater recruitment of hip extensors during the middle to late phases of squat-to-stand movement. In addition, there was a decrease in recruitment of lumbar extensors during the middle phase, but an increase during the late phase, of the squat-to-stand task in their lumbar extensors after 15 min squatting. This distinctive pattern of joint kinematics and recruitment of the key lumbopelvic muscles shown in those with chronic nonspecific LBP after prolonged squatting provides new knowledge that helps unravel the possible mechanisms which may increase the risk of development and/or aggravation of lumbar dysfunctions for people who work in prolonged deep squatting postures. The present findings have also provided the preliminary but supportive evidence for emphasizing training of respective groups of muscles, for example, the primary hip extensors, when addressing the modified recruitment pattern of the muscles in the lumbopelvic region for people with chronic LBP.

## Figures and Tables

**Table 1:** Group demographics.

Demographics	LBP Group (*n* = 8)	AS Group (*n* = 8)	*p*-Value ^1^
Age	24.50 ± 2.62	25.38 ± 2.88	0.535
Height (m)	1.72 ± 0.05	1.70 ± 0.08	0.626
Weight (kg)	69.94 ± 7.68	66.85 ± 15.48	0.621
BMI (m/kg^2^)	23.65 ± 2.71	23.11 ±5.43	0.807
History of chronic LBP (month)	23.75 ± 19.26	N/A ^2^	N/A
VAS before experiment (0–10)	0–0.5	0	N/A

Notes: ^1^ By independent *t*-test; ^2^ not applicable. AS: asymptomatic; LBP: lower back pain; VAS: visual analogue scale.

**Table 2:** Lumbar spine, hip joint angles in simulated deep squatting position.

Joint Angle	LBP Group	AS Group	*p*-Value ^1^
Lumbar (°)	42 ± 5	33 ± 16	0.158
(R) hip (°)	104 ± 15	102 ± 11	0.835
(L) hip (°)	101 ± 15	100 ± 13	0.918
Mean of hip flexion angles ^2^ (°)	102 ± 15	101 ± 12	0.535
Total flexion angle (°)	144 ± 13	134 ± 14	0.146

Notes: ^1^ By independent *t*-test; ^2^ mean flexion angle of both hips.

**Table 3:** The lumbar erector spinae (ES) activity level (percent maximal voluntary contraction, %MVC) during simulated deep squatting position.

LBP Subject	1	2	3	4	5	6	7	8
(R) ES	1.79%	1.97%	2.21%	2.92%	3.95%	3.99%	4.01%	9.70%
(L) ES	1.64%	1.80%	2.56%	2.87%	3.49%	3.71%	4.64%	11.25%
AS Subject	1	2	3	4	5	6	7	8
(R) ES	0.90%	1.40%	1.75%	2.93%	3.20%	3.92%	7.97%	12.48%
(L) ES	1.02%	1.18%	2.62%	4.26%	4.57%	6.63%	7.89%	8.22%

## References

[1] Sterud T., Tynes T. (2013). Work-related psychosocial and mechanical risk factors for low back pain: A 3-year follow-up study of the general working population in Norway. Occup. Environ. Med..

[2] Solomonow M., Zhou B.H., Baratta R.V., Burger E. (2003). Biomechanics and electromyography of a cumulative lumbar disorder: Response to static flexion. Clin. Biomech..

[3] Van Vuuren B.J., Becker P.J., van Heerden H.J., Zinzen E., Meeusen R. (2005). Lower back problems and occupational risk factors in a South African steel industry. Am. J. Ind. Med..

[4] Pal A., De S., Sengupta P., Maity P., Dhara P.C. (2015). Evaluation of work related musculoskeletal disorder and postural stress among female potato cultivators in West Bengal, India. Ergon. SA.

[5] Jia N., Li T., Hu S., Zhu X., Sun K., Yi L., Zhang Q., Luo G., Li Y., Zhang X. (2016). Prevalence and its risk factors for low back pain among operation and maintenance personnel in wind farms. BMC Musculoskelet. Disord..

[6] Park S.-A., Shoemaker C.A. (2009). Observing body position of older adults while gardening for health benefits and risks. Act. Adapt. Aging.

[7] Panjabi M.M. (1992). The Stabilizing System of the Spine. Part I. Function, Dysfunction, Adaptation, and Enhancement. Clin. Spine Surg..

[8] Mueller M.J., Maluf K.S. (2002). Tissue Adaptation to Physical Stress: A Proposed ‘Physical Stress Theory’ to Guide Physical Therapist Practice, Education, and Research. Phys. Ther..

[9] Bergmark A. (1989). Stability ofthe lumbar spine. A study in mechanical engineering. Acta Orthop. Scand. Suppl..

[10] Cholewicki J., McGill S.M. (1996). Mechanical stability of the in vivo lumbar spine: Implications for injury and chronic low back pain. Clin. Biomech..

[11] Callaghan J.P., Dunk N.M. (2002). Examination of the flexion relaxation phenomenon in erector spinae muscles during short duration slumped sitting. Clin. Biomech..

[12] Shirado O., Ito T., Kaneda K., Strax T.E. (1995). Flexion-relaxation phenomenon in the back muscles. A comparative study between healthy subjects and patients with chronic low back pain. Am. J. Phys. Med. Rehabilit..

[13] Solomonow M. (2012). Neuromuscular manifestations of viscoelastic tissue degradation following high and low risk repetitive lumbar flexion. J. Electromyogr. Kinesiol..

[14] Shin G., D’Souza C., Liu Y.-H. (2009). Creep and Fatigue Development in the Low Back in Static Flexion. Spine.

[15] Abboud J., Nougarou F., Descarreaux M. (2016). Muscle Activity Adaptations to Spinal Tissue Creep in the Presence of Muscle Fatigue. PLoS ONE.

[16] Sánchez-Zuriaga D., Adams M.A., Dolan P. (2010). Is Activation of the Back Muscles Impaired by Creep or Muscle Fatigue?. Spine.

[17] Sung P.S. (2013). A compensation of angular displacements of the hip joints and lumbosacral spine between subjects with and without idiopathic low back pain during squatting. J. Electromyogr. Kinesiol..

[18] Eungpinichpong W., Buttagat V., Areeudomwong P., Pramodhyakul N., Swangnetr M., Kaber D., Puntumetakul R. (2013). Effects of restrictive clothing on lumbar range of motion and trunk muscle activity in young adult worker manual material handling. Appl. Ergon..

[19] Schelldorfer S., Ernst M.J., Rast F.M., Bauer C.M., Meichtry A., Kool J. (2015). Low back pain and postural control, effects of task difficulty on centre of pressure and spinal kinematics. Gait Posture.

[20] Bauer C.M., Rast F.M., Ernst M.J., Oetiker S., Meichtry A., Kool J., Rissanen S.M., Suni J.H., Kankaanpää M. (2015). Pain intensity attenuates movement control of the lumbar spine in low back pain. J. Electromyogr. Kinesiol..

[21] Bauer C.M., Rast F.M., Ernst M.J., Kool J., Oetiker S., Rissanen S.M., Suni J.H., Kankaanpää M. (2015). Concurrent validity and reliability of a novel wireless inertial measurement system to assess trunk movement. J. Electromyogr. Kinesiol..

[22] Hermens H.J., Freriks B., Disselhorst-Klug C., Rau G. (2000). Development of recommendations for SEMG sensors and sensor placement procedures. J. Electromyogr. Kinesiol..

[23] Hermens H.J., Merletti R., Freriks B. (1999). SENIAM European Recommendations for Surface Electromyography.

[24] Arokoski J.P., Valta T., Kankaanpää M., Airaksinen O. (2004). Activation of lumbar paraspinal and abdominal muscles during therapeutic exercises in chronic low back pain patients. Arch. Phys. Med. Rehabilit..

[25] De Ridder E.M., Van Oosterwijck J.O., Vleeming A., Vanderstraeten G.G., Danneels L.A. (2013). Posterior muscle chain activity during various extension exercises: An observational study. BMC Musculoskelet. Disord..

[26] Maffiuletti N.A., Lepers R. (2003). Quadriceps Femoris Torque and EMG Activity in Seated versus Supine Position. Med. Sci. Sports Exerc..

[27] Olson M.W., Li L., Solomonow M. (2004). Flexion-relaxation response to cyclic lumbar flexion. Clin. Biomech..

[28] Naserkhaki S., Jaremko J.L., El-Rich M. (2016). Effects of inter-individual lumbar spine geometry variation on load-sharing: Geometrically personalized Finite Element study. J. Biomech..

[29] Jin S., Ning X., Mirka G.A. (2012). An algorithm for defining the onset and cessation of the flexion-relaxation phenomenon in the low back musculature. J. Electromyogr. Kinesiol..

[30] Solomonow M., Baratta R.V., Zhou B.H., Burger E., Zieske A., Gedalia A. (2003). Muscular dysfunction elicited by creep of lumbar viscoelastic tissue. J. Electromyogr. Kinesiol..

[31] Marras W.S., Lavender S.A., Leurgans S.E., Fathallah F.A., Ferguson S.A., Gary Allread W., Rajulu S.L. (1995). Biomechanical risk factors for occupationally related low back disorders. Ergonomics.

[32] Marras W.S., Parakkat J., Chany A.M., Yang G., Burr D., Lavender S.A. (2006). Spine loading as a function of lift frequency, exposure duration, and work experience. Clin. Biomech..

[33] Granata K.P., Rogers E., Moorhouse K. (2005). Effects of static flexion-relaxation on paraspinal reflex behavior. Clin. Biomech..

[34] Granata K.P., Slota G.P., Wilson S.E. (2004). Influence of Fatigue in Neuromuscular Control of Spinal Stability. Hum. Factor..

[35] Hart J.M., Fritz J.M., Kerrigan D.C., Saliba E.N., Gansneder B.M., Ingersoll C.D. (2006). Quadriceps Inhibition After Repetitive Lumbar Extension Exercise in Persons with a History of Low Back Pain. J. Athl. Train..

[36] Wilson J., Ferris E., Heckler A., Maitland L., Taylor C. (2005). A structured review of the role of gluteus maximus in rehabilitation. J. Physiother..

[37] Leinonen V., Kankaanpää M., Airaksinen O., Hãnninen O. (2000). Back and hip extensor activities during trunk flexion/extension: Effects of low back pain and rehabilitation. Arch. Phys. Med. Rehabilit..

[38] Vogt L., Pfeifer K., Banzer W. (2003). Neuromuscular control of walking with chronic low-back pain. Man. Ther..

[39] Pries E., Dreischarf M., Bashkuev M., Putzier M., Schmidt H. (2015). The effects of age and gender on the lumbopelvic rhythm in the sagittal plane in 309 subjects. J. Biomech..

[40] Kramer M., Schmid I., Sander S., Högel J., Eisele R., Kinzl L., Hartwig E. (2003). Guidelines for the intramuscular positioning of EMG electrodes in the semispinalis capitis and cervicis muscles. J. Electromyogr. Kinesiol..

[41] Delitto R.S., Rose S.J. (1992). An Electromyographic Analysis of Two Techniques for Squat Lifting and Lowering. Phys. Ther..

[42] Hayashi S., Katsuhira J., Matsudaira K., Maruyama H. (2016). Effect of pelvic forward tilt on low back compressive and shear forces during a manual lifting task. J. Phys. Ther. Sci..

